# Treating Mooren's Ulcer - Squeezing Water From a Stone

**DOI:** 10.7759/cureus.12248

**Published:** 2020-12-23

**Authors:** Aamir Husain, Amna Saleem, Zain Ali Zaidi, Zohra Kazmi, Uzzam Ahmed Khawaja

**Affiliations:** 1 Opthalmology, Jinnah Medical and Dental College, Karachi, PAK; 2 Neurological Surgery, General Surgery, Jinnah Medical and Dental College, Karachi, PAK; 3 Internal Medicine, Jinnah Medical and Dental College, Karachi, PAK; 4 Medicine, Jinnah Medical and Dental College, Karachi, PAK; 5 Clinical and Translational Research, Larkin Community Hospital, South Miami, USA

**Keywords:** mooren's ulcer, topical treatment, tarsorrhaphy, immunosuppression, idiopathic

## Abstract

Mooren's ulcer, a rare ophthalmic disease, presents clinically as a painful, chronic, peripheral corneal ulceration of unknown etiology with some autoimmune origin evidence. It begins with an intense limbal inflammation, leaving behind an opaque cornea. If left untreated, progressive damage and corneal degeneration can lead to permanent loss of vision. Herein, we present a classic case of Mooren's ulcer in the right eye of a 60-year-old male patient with no known comorbid condition. No underlying systemic disorder being the rarity in our case, the cause remains idiopathic. The patient was previously diagnosed with having Mooren's ulcer in his left eye 10 years ago. Despite multiple topical treatments and surgical interventions, there was a complete loss of vision. He presented exaggerated manifestations, including pain, redeye, watery eye, photophobia, and the progressive decline of vision. A combination of multiple pharmacological and surgical interventions, including lateral tarsorrhaphy, amniotic membrane grafting, conjunctival flap, and scleral patch graft, was tried to ameliorate the affected eye but failed to salvage the eye permanently.

## Introduction

Mooren's ulcer is a severe, sustained peripheral ulcerative keratitis beginning from the peripheral cornea, initially spreading circumferentially, and ending up involving the center of the cornea [1]. Initially, it starts as an intense limbal inflammation, followed by swelling in the episclera and conjunctiva, and may damage the corneal stroma [2]. Around 25% of the affected patients present with bilateral involvement; however, a unilateral presentation can also be observed [3]. Even though past studies have suggested an autoimmune origin, certain infections, hepatitis C, trauma, or corneal surgeries could play a role in the pathogenesis [2]. Since there might be an autoimmune mechanism involved, steroids and other immunosuppressive therapies have demonstrated an effective long-term response. Surgical interventions such as conjunctival flap therapy or keratoplasty are commonly performed procedures [4]. However, the disease is usually aggressive, despite optimal treatment measures and attempts to salvage the affected tissue.

Our treatment regimen included multiple topical medications and the surgical procedure of lateral tarsorrhaphy, which significantly cured the patient temporarily. The purpose of tarsorrhaphy was to heal or at least protect the cornea from exposure and allow the tear film to enhance the healing mechanism [5]. However, after the impact of tarsorrhaphy decreased significantly, we tried to treat him with immunosuppressants along with corticosteroids to restrict the damage caused by any autoimmune insult. Since the patient's condition was progressively deteriorating, we had to proceed with multiple surgeries at different sets of conditions, including amniotic membrane grafting, conjunctival flap, and scleral patch graft. Despite many attempts, the manifestation remained untreatable. We intend to apprise the world of ophthalmology regarding additional treatment options for the calamitous manifestation through this case report.

## Case presentation

**Figure 1:**
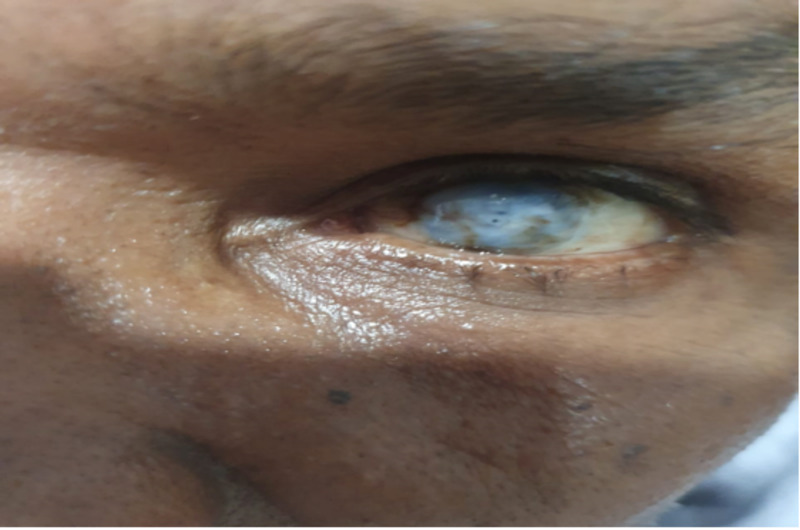
Mooren's ulcer in the left eye.

**Figure 2:**
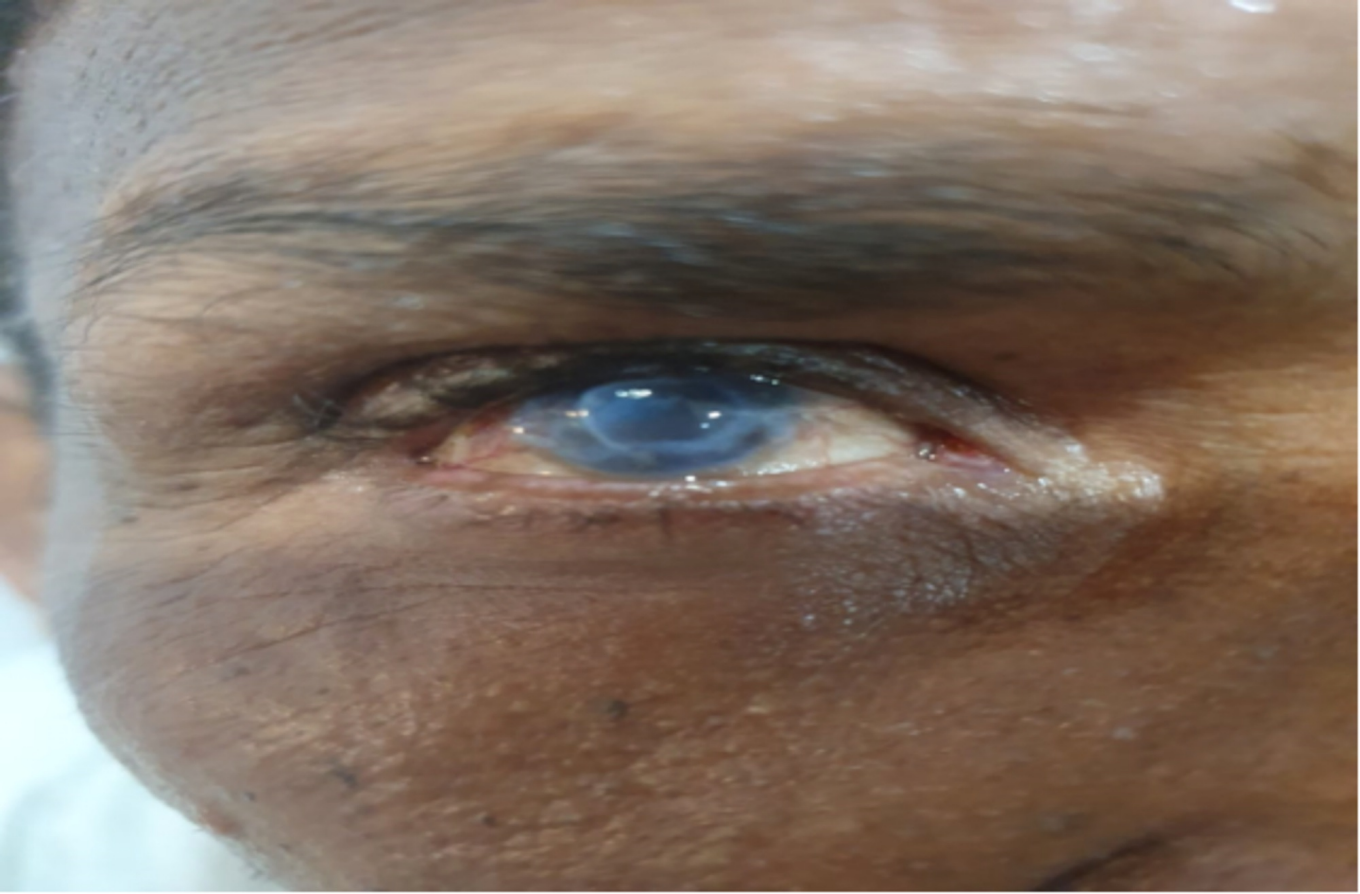
Mooren's ulcer in the right eye.

A 60-year-old male patient presented to our ophthalmology out-patient department (OPD) with chief complaints of severe pricking pain, foreign body sensation along with redness, watery eyes, photophobia, and progressive blurring of vision in his right eye for the past one week. Previously, he was diagnosed with Mooren's ulcer in his left eye in 2009 (Figure 1). At that time, despite undergoing several treatment regimens, including a conjunctival flap procedure and the use of multiple topical medications, the ulcer did not heal, and the patient ended up having complete loss of vision from his left eye. In 2019, the patient observed the re-emergence of similar symptoms in his right eye (Figure 2) and presented with sharp pricking pain, which aggravated on exposure to light to such an extent that he was unable to open his eyes. The patient also complained of serous discharge and redness in the affected eye. The examination revealed visual acuity of 6/9 right eye (OD) and 6/9 left eye (OS). Further ocular examination exhibited a perforating Mooren's ulcer at 3 o'clock to 9 o'clock position, extending from the nasal to the temporal cornea involving the peripheries, forming a 180-degree crescent-shaped limbal gutter. There was grey-white opacification in almost the whole of the cornea circumferentially, sparing its superior most part, and the ulcer had not yet invaded the central portion. Moreover, there was no evidence of associated scleritis.

His systemic examinations were unremarkable. There was no history of blood transfusions and no significant drug history. However, he was a cigarette smoker for 15-20 years and smoked four to five cigarettes per day. On lab investigations his complete blood count (CBC) was normal, creatinine was 1.59 mg/dL (0.6 - 1.2 mg/dL), urea levels were 33 mg/dL (7 - 18 mg/dL). Liver function tests (LFTs), urine DR (detailed report) and serum electrolytes were normal. His random blood sugar (RBS) was 108 mg/dL (<120 mg/dL), hence ruling out diabetes mellitus as a possible comorbid. His hepatitis B and C reactive antigens were negative, and there was no significant finding in any of the labs we conducted, thereby the cause was completely idiopathic.

Initially, during the patient's first visit, he was prescribed one to two drops of levobunolol 0.25% twice a day (BID), cyclopentolate 1%, dexamethasone, and chloramphenicol drops. The patient administered these drugs for around 2.5 months that healed the eye between 3 o'clock till 7 o'clock position, after which he was prescribed lateral tarsorrhaphy. The goal of tarsorrhaphy was to protect the cornea from excessive tearing and reduce exposure symptoms, especially in the unhealed area. His postoperative treatment included fluorometholone 0.25%, polyacrylic acid 0.1 mg (after every eight hours), cyclosporin drops 0.05% after every 12 hours, polyvinyl alcohol, ciprofloxacin eye drops 0.3% three times a day, diclofenac potassium oral 50 mg BID, and amlodipine/valsartan 10/160 mg OD. The surgery provided temporary relief but, unfortunately, was ineffective in the long term. He was kept on methotrexate 10 mg once a week. To reconstruct the cornea's impregnable destruction, amniotic membrane grafting was performed to build thickness in the defect and promote faster healing. Impersonating the outcomes of previous surgery, amniotic membrane grafting did not exhibit the desired results, and after few weeks, a conjunctival flap was conducted followed by a scleral patch graft to reinstate the unification of the chronically compromised corneal surface. This surgery was thought to be most definitive to reduce ocular inflammation, improve ulcer healing, and provide mechanical support. After these surgeries, postoperative treatment included oral ciprofloxacin 250 mg BID, ibuprofen 200 mg BID, dexamethasone sodium and chloramphenicol drops five times a day, and Polyfax eye ointment (bacitracin zinc and polymyxin B sulfate) used as needed (SOS). Unfortunately, even after endeavoring a combination of multiple surgeries and different treatment regimens, the concerted effect was not fruitful for the permanent healing of this recalcitrant ulcer.

## Discussion

Mooren's ulcer is a severe and aggressive autoimmune disease of unknown etiology with the cornea's involvement and calamitous destruction. If left untreated, it can progress to perforation and even complete loss of vision [6]. According to the literature review, it has been suggested that Mooren's ulcer is more or less associated with Hepatitis C, trauma, and parasitic infections and that it is an autoimmune pathology against the cornea [2,7], yet there is no firm evidence to prove this association. Our case was anomalous since all the systemic examinations and lab findings evinced negligible results, signifying an idiopathic origin. Since the disease itself is a rare disorder, the treatment remains challenging too, with an unknown definitive etiology.

The past treatments included immunosuppressants, interferons-alfa2A, corticosteroid therapy, bandage contact lens, conjunctival resection, and corneoscleral graft [1,2,6]. Deriving an ideal medicinal regimen for this severe corneal ulcer is an intensely strenuous task. In our case, we implemented multiple techniques to cure and salvage the affected eye. We initiated the treatment with topical drugs and steroids to limit the damage and provide relief to the inflamed areas of the eye. Steroid therapy is recognized as a worthy option due to its efficacy and combative control on corneal destruction [3]. It successfully mended the ulcer such that it was healed from a 3 o'clock to a 7 o'clock position. To repair the cornea's unhealed segment, the surgical process of lateral tarsorrhaphy was performed, a procedure to seal a part of the eye by surgically connecting the upper and lower eyelids, thereby minimizing the friction of eyelids with the ocular surface [5].

Previously tarsorrhaphy has successfully treated cases of inadequate eyelid coverage, which causes exposure of cornea, Grave's ophthalmopathy, and even in cases of Bell's palsy, which causes facial nerve dysfunction [8]. The aim of lateral tarsorrhaphy was to repair and protect the ulcerated part of the cornea and reduce exposure, tear film evaporation and allow the natural tears to heal the diseased part on its own. Our target was somewhat accomplished since our treatment strategy proved beneficial in healing the ulcer and the symptoms regressed. However, after a month, there was a relapse of symptoms, and the regimen failed to cease terrible outcomes of the disease in the long term. Due to intense exposure after undoing the stitches, there was recurrent inflammation with intense corneal epithelial defects and deteriorating vision. The situation became so adverse that it led to the initiation of corneal melting. To avoid scaring and reverse the symptoms, we introduced another surgical procedure of amniotic membrane grafting. Previously, it has been proven to have anti-inflammatory and anti-scarring properties with favorable outcomes and a tendency to promote faster healing.

In 1914, de Rotth introduced amniotic membrane transplantation in the world of ophthalmology after achieving partial success in treating conjunctival epithelial defects [9]. Postoperative measures for the patient included immunosuppressant therapy to reduce the risk of any autoimmune damage. Unfortunately, this treatment was not successful yet again, and there was a drastic decline in the ulcer's healing. To rectify and salvage the devastating ulcer, the last measure taken was a conjunctival flap followed by a scleral patch graft. This procedure is well known in the world of ophthalmology for being beneficial in the treatment of corneal ulceration and reinforced cataract wounds, herpetic ulcers, neuroparalytic keratitis, relapsing erosions, bullous keratopathies, and even graft rejection [10]. The conjunctival flap has been demonstrated to be a simple, well-supported, short-term treatment for the management of corneal perforation or impending perforation in infective corneal ulcers [11-13]. It also provides mechanical support for the corneal ulceration to heal. The conjunctival flap interventions are commonly admired in the persistent corneal ulcers after the failure of various approaches, including the few which were performed on our patient. However, this last attempt to salvage the eye was also not successful in our patient, whose condition deteriorated and ended up with complete blindness.

## Conclusions

Bilateral Mooren's ulcer mandates prompt treatment as visual morbidity remains significant. As conclusive from our case, topical treatment and different surgical procedures followed by postoperative immunosuppressive therapy did not prove to be the mainstay of treatment. More research and trials need to be conducted to permanently derive a proper therapeutic regimen for curing this destructive disease.
